# Ascertaining the Francophone population in Ontario: validating the language variable in health data

**DOI:** 10.1186/s12874-024-02220-7

**Published:** 2024-04-27

**Authors:** Ricardo Batista, Amy T. Hsu, Louise Bouchard, Michael Reaume, Emily Rhodes, Ewa Sucha, Eva Guerin, Denis Prud’homme, Douglas G. Manuel, Peter Tanuseputro

**Affiliations:** 1 Institut du Savoir Montfort, Ottawa, ON Canada; 2 ICES uOttawa, Ottawa, ON Canada; 3 Clinical Epidemiology Program, Ottawa Hospital Research Institute, Ottawa, ON Canada; 4 Université de Moncton, Moncton, New Brunswick, Canada; 5 Elizabeth Bruyère Research Institute, Ottawa, ON Canada; 6 School of Social and Anthropological Studies, University of Ottawa, Ottawa, ON Canada; 7 University of Manitoba, Winnipeg, MB Canada; 8 Faculty of Medicine, University of Ottawa, Ottawa, ON Canada; 9 Statistics Canada, Ottawa, ON Canada; 10 Institut du Savoir Montfort, ICES and Ottawa Hospital Research Institute, 1053 Carling Ave Box 693, 2-006 Admin Services Building, Ottawa, ON K1Y 4E9 Canada

**Keywords:** Validity, Linguistic variables, Administrative health data, Case ascertainment, Francophones

## Abstract

**Background:**

Language barriers can impact health care and outcomes. Valid and reliable language data is central to studying health inequalities in linguistic minorities. In Canada, language variables are available in administrative health databases; however, the validity of these variables has not been studied. This study assessed concordance between language variables from administrative health databases and language variables from the Canadian Community Health Survey (CCHS) to identify Francophones in Ontario.

**Methods:**

An Ontario combined sample of CCHS cycles from 2000 to 2012 (from participants who consented to link their data) was individually linked to three administrative databases (home care, long-term care [LTC], and mental health admissions). In total, 27,111 respondents had at least one encounter in one of the three databases. Language spoken at home (LOSH) and first official language spoken (FOLS) from CCHS were used as reference standards to assess their concordance with the language variables in administrative health databases, using the Cohen kappa, sensitivity, specificity, positive predictive value (PPV), and negative predictive values (NPV).

**Results:**

Language variables from home care and LTC databases had the highest agreement with LOSH (kappa = 0.76 [95%CI, 0.735–0.793] and 0.75 [95%CI, 0.70–0.80], respectively) and FOLS (kappa = 0.66 for both). Sensitivity was higher with LOSH as the reference standard (75.5% [95%CI, 71.6–79.0] and 74.2% [95%CI, 67.3–80.1] for home care and LTC, respectively). With FOLS as the reference standard, the language variables in both data sources had modest sensitivity (53.1% [95%CI, 49.8–56.4] and 54.1% [95%CI, 48.3–59.7] in home care and LTC, respectively) but very high specificity (99.8% [95%CI, 99.7–99.9] and 99.6% [95%CI, 99.4–99.8]) and predictive values. The language variable from mental health admissions had poor agreement with all language variables in the CCHS.

**Conclusions:**

Language variables in home care and LTC health databases were most consistent with the language often spoken at home. Studies using language variables from administrative data can use the sensitivity and specificity reported from this study to gauge the level of mis-ascertainment error and the resulting bias.

**Supplementary Information:**

The online version contains supplementary material available at 10.1186/s12874-024-02220-7.

## Introduction

In recent years, studies have provided evidence for the existence of health disparities across linguistic groups in Canada [1, 2]. However, most studies relied on census and survey data to examine the disparities by language characteristics [3–5]. Administrative health databases are widely used to assess health and health care disparities; but the availability and quality of the language information is a barrier to performing health research on linguistic groups in Canada [6, 7]. Methodological challenges have hindered further research on the relationship between linguistic factors and health outcomes. Quality issues derived from collection methods, type of language recorded, and access to data prevent researchers from further exploring how linguistic factors are impacting health care and outcomes [8–10]. Some studies have used language variables collected in healthcare databases; however, since their validity has never been formally assessed, the use of these variables has been limited and has generated conflicting results [8, 9, 11]. 

### Language variables and linguistic groups

Linguistic groups are usually defined through language variables, either by a single variable that represents a simple linguistic concept (e.g., mother tongue, language most often spoken at home [LOSH], language of preference, etc.) or a combination of multiple variables (e.g., First Official Language Spoken [FOLS], which is derived from the Mother Tongue, Knowledge of Canadian Official Languages and LOSH) [12]. Many of these language variables are routinely collected in the census and. Canadian Community Health Survey [CCHS]) in Canada and less often in administrative health databases.

Mother tongue, LOSH and more increasingly FOLS, are the language variables most commonly used in Canada to define and describe the characteristics of linguistic groups and to conduct comparative analyses in many studies, including those focusing on healthcare [9, 12–15]. FOLS, which is defined within the framework of the Official Languages Act and represents a combination of several language variables, is increasingly being used in analyses and reports by Statistics Canada [14, 16, 17]. FOLS is valuable for research purposes because it establishes linguistic groups denoting Canada’s two official languages (English and French) while also including persons whose mother tongue is neither English nor French but who use one or both of these languages on a regular basis. Francophones are a linguistic minority outside Quebec. In Ontario, francophones make up about 4% of the population and research shows that francophone Ontarians face important health inequalities [5, 18, 19], but most of the analyses use survey data and only a few studies have used health data to identify the linguistic groups [11, 20–22]. However, no previous study has examined the validity of the language information in administrative health data. Thus, we used several health databases from Ontario to assess its validity to identify francophones in health research.

This study sought to determine the ability to ascertain Francophones in Ontario using administrative health databases. Specifically, we assessed measures validity derived from language variables in administrative health databases to identify francophones, against a national survey standard, the CCHS, and determined the language concept captured by these variables.

## Methods

The study used a data linkage of Ontario combined samples of the CCHS cycles 1.1 (2000–2001) to 2012 that were securely linked to three administrative health databases using anonymized and unique encoded identifiers and analyzed in a secure environment at ICES (https://www.ices.on.ca/; formerly Institute for Clinical Evaluative Sciences).

### Data sources

The study population included Ontario respondents to the CCHS cycle 1.1 (2000–2001) to 2012 cycle, 20 years and older who: (1) agreed to have their survey responses shared with the provinces and linked to their health care data (approximately 85% of participants) and (2) were eligible for Ontario’s universal health insurance plan (OHIP). The CCHS is a cross-sectional national representative survey that collects information related to health status, health care utilization and health determinants of the Canadian population aged 12 years or older living in private dwellings in all provinces and territories. To the best of our knowledge, there are no systematic differences between participants in CCHS who provided consent to link their data and those who did not.

Thus, for creating the study dataset, the CCHS samples for Ontario (cycle 1.1 [2000–2001], cycle 2.1 [2003], cycle 3.1 [2005], cycle 4.1 [2007], 2009–2010 and 2011–2012) were combined. Then, the CCHS combined dataset was linked to three health databases that contain language information: the Continuing Care Reporting System (CCRS), which collects population-based resident information of patients receiving 24-hour nursing care in publicly funded residential long-term care; the Home Care Reporting System (HCRS), which comprises data using the Resident Assessment Instrument-Home Care (RAI-HC), which collects information on adults expected to receive home care services for at least six months; and the Ontario Mental Health Reporting System (OMHRS), which collects data on patients admitted to inpatient mental health services. Eligible participants were identified using OHIP and Registered Persons Database (RPDB) and were linked over the same period covered by the survey. Each dataset used in the study is described in Appendix 1.

### Reference standard

Although there is no consensus regarding a reference standard for evaluating the quality of administrative data [23], numerous studies have used data from national representative surveys that provide accurate estimates of population characteristics, such as the CCHS, to validate administrative data in ascertaining chronic conditions (e.g., diabetes, hypertension, osteoporosis) [24–31]. Language variables collected in self-report surveys (e.g., Census, CCHS) are more explicitly defined than administrative databases. The CCHS includes original language variables (e.g., mother tongue, LOSH, and knowledge of official languages) and derived variables, such as FOLS, which are based on two or more language variables. Despite minor modifications to variable definitions since the inception of the CCHS, these variables provide accurate estimates of the linguistic characteristics of the Canadian population [19, 32, 33]. 

Given the validity of national representative surveys conducted by Statistics Canada, we used the language variables from the CCHS, LOSH, an original variable collected in the survey and FOLS, which is a derived variable from the knowledge of official languages, mother tongue, and LOSH [34] as the reference standard measures to assess the capacity of health data to ascertain the French-speaking population. The levels of non-response for the language variables in CCHS was low across cycles (< 5%), ranging from 0.2 to 2.7%. The levels of missing values in health data were also lower than 5%. We did not exclude the records with missing values for these variables and made no imputations.

**Table:** 

From CCHS:	From administrative health databases (language variable label):
- Mother tongue	- HCRS (Primary Language)
- Language spoken most often at home (LOSH)	- CCRS (Primary language spoken at home on a regular basis)
- Knowledge of official languages	- OMHRS (Language)
- Language of conversation	
- Language of interview	
- Language of preference	
- Language spoken to a doctor	
- First official language spoken (FOLS)	

CCRS: Continuing Care Reporting System, HCRS: Home Care Reporting System, OMHRS: Ontario Mental Health Reporting System, CCHS: Canadian Community Health Survey

### Administrative data and language information

The three administrative health databases (CCRS, HCRS and OMHRS) containing language information were used to identify Francophones. Without a clear and specific language definition, administrative health databases may be subject to interviewer bias (i.e., the interviewer may assume the respondent’s language without explicitly asking for this information). Thus, the language variables from CCHS were used as the reference standard to validate the language variables in the health data. There are several language variables included in the survey (see Appendix 2), but LOSH and FOLS were used for the validity analysis.

The language variables Mother tongue, LOSH and language of conversation in CCHS allowed to derive the Knowledge of official languages and FOLS, following Statistics Canada’s definition [34]. Details on the collection of language variables are provided in Appendix 2.

Although it is possible to make population estimates using CCHS survey weights, in this study we reported unweighted values, which were used to perform the individual data linkage and the analyses.

### Analysis

Descriptive analyses of the language variables in all databases were performed. First, a frequency analysis of all language variables was conducted, and the proportion of participants in each linguistic group was reported. We provide a covariate description of the sample stratified by language group (i.e. francophones) and by age group, sex, rural/urban area of residence, marital and immigrant status, education and income levels. Second, the linked data set was used to evaluate the concordance of the language variables in identifying francophones by performing an agreement analysis using Cohen’s kappa coefficient, which is a widely used measure of concordance between assessors and indicates the proportion of agreement beyond that expected by chance [35]. The levels of agreement for kappa were considered poor (κ < 0.20), fair (κ = 0.20 to 0.39), moderate (κ = 0.40 to 0.59), good (κ = 0.60 to 0.79), or very good (κ = 0.80 to 1.00) [25, 36]. Next, validity analyses were performed to determine the language concept captured by the language variables in administrative data. The validity of the language variables in administrative health data for identifying francophones was assessed by calculating the sensitivity, specificity, positive predictive value (PPV), and negative predictive value (NPV) [36, 37] using FOLS and LOSH as reference standards. All analyses were conducted using SAS 9.4 (SAS Institute, Inc., Cary, NC).

This project was approved by ICES’ Privacy and Compliance Office. ICES is a prescribed entity under Sect. 45 of Ontario’s Personal Health Information Protection Act, which does not require review by a Research Ethics Board.

## Results

**Fig. 1:**
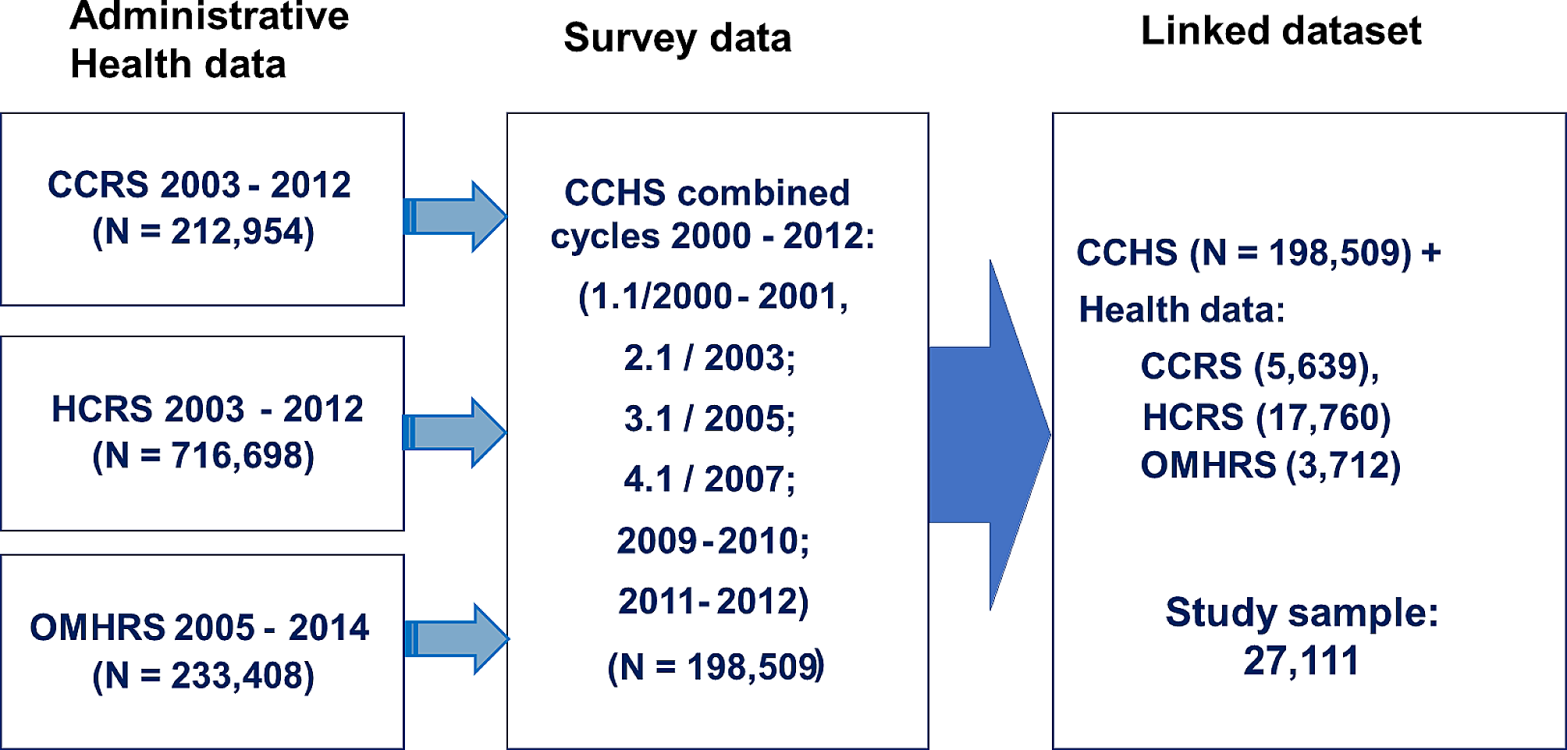
Sample size from each data source and linked sample. CCRS: Continuing Care Reporting System. HCRS: Home Care Reporting System. OMHRS: Ontario Mental Health Reporting System. CCHS: Canadian Community Health Survey

The combined CCHS sample consisted of 198,509 respondents, which were individually linked to their provincial health card number that allowed individual linkage to the administrative databases, resulting in Ontarians within CCRS (including 212,954 individuals)), HCRS (*n* = 716,698 individuals) and OMHRS (*n* = 233,408 individuals). The linked dataset consisted of individuals who participated in at least one cycle of the CCHS cycle and who were captured in at least one of the three administrative health databases for the timespan of the CCHS cycles (2000–2012). The final study sample consisted of 27,111 CCHS respondents who received home care services (HCRS) or long-term care services (CCRS) or were admitted to an inpatient mental health service (OMHRS) (Fig. 1). A summary of the characteristics of these databases by language group is provided in Table S1 in supplementary material.

Table 1 presents the unweighted frequencies for the characteristics of the 198,509 respondents from the combined CCHS cycles, and the characteristics of francophones identified by FOLS and LOSH (a weighed sample is presented in Table S2, Appendix 3).

**Table 1:** Sociodemographic characteristics of Ontarians who completed CCHS Cycle 1.1 (2000–2001) to CCHS 2012 (unweighted frequencies)

	Total population		French-speakers			
			by FOLS*		by LOSH	
	(*n* = 198,509)		(*n* = 10,036)		(*n* = 6,040)	
	n	%	*n*	*%*	*n*	*%*
**Age group**						
< 18	18,940	9.5	684	6.8	479	7.9
18–49	87,619	44.1	3,941	39.3	2382	39.4
50–59	30,629	15.4	1,905	19.0	1101	18.2
60–69	28,435	14.3	1,783	17.8	1066	17.6
70–79	21,501	10.8	1,207	12.0	710	11.8
80–89	10,340	5.2	472	4.7	272	4.5
90+	1,045	0.5	44	0.4	30	0.5
**Sex**						
Male	90,779	45.7	4,236	42.2	2500	41.4
Female	107,730	54.3	5,800	57.8	3540	58.6
**Urban and Rural Areas**						
Urban	154,269	77.7	7,366	73.4	4260	70.5
Rural	44,240	22.3	2,670	26.6	1780	29.5
**Immigrant**						
Yes	38,441	19.4	448	4.5	307	5.1
No	159,689	80.6	9,583	95.5	5731	94.9
**Marital status**						
Married	91,967	46.4	4,618	46.1	2757	45.6
Common-law	11,256	5.7	921	9.2	528	8.7
Widowed	19,436	9.8	1,159	11.6	681	11.3
Separated	6,750	3.4	452	4.5	251	4.2
Divorced	12,343	6.2	636	6.3	333	5.5
Single, never married	56,642	28.6	2,243	22.4	1487	24.6
**Highest level/education**						
< Second. School Grad.	52,051	26.4	3,041	30.5	1942	32.2
Secondary School Grad.	35,645	18.1	1,480	14.8	837	13.9
Some Post-secondary	13,172	6.7	573	5.7	297	4.9
Post-Secondary Grad.	96,350	48.9	4,889	49.0	2928	48.5
**Household income**						
Quintile 1	23,228	18.9	700	20.6	906	15.0
Quintile 2	23,328	18.9	678	17.9	784	13.0
Quintile 3	24,700	20.1	692	18.9	814	13.5
Quintile 4	25,006	20.3	690	19.9	906	15.0
Quintile 5	26,869	21.8	784	22.7	1015	16.8

CCHS: Canadian Community Health Survey, FOLS: First official language spoken

^*^ Redefined for cycles 2003 to 2009, based on Statistics Canada definition of FOLS introduced in 2011 (Ref. 25)

Within the study sample, 6.3% were French speakers by mother tongue, 6.0% were identified as Francophone by FOLS, and 3.6% reported using French as the language often spoken at home (Table 2 and Table S3, Appendix 3). Less than 2% of respondents conducted the interview in French or indicated French as their preferred language for the interview. Even fewer respondents (1.8%) reported speaking French with their doctor. Based on the language variables in administrative health databases, long-term care data (CCRS) identified the largest proportion of French speakers (3.2%), followed by home care data using the HCRS (2.8%).

**Table 2:** Frequency of Francophones by type of language variable from each data source

Language variables - CCHS survey data	French speakers (%)
*(Total unweighted sample size from CCHS Cycle 1.1 (2000–2001) to CCHS 2012, n = 198,509)*	
Mother tongue	12,530 (6.3%)
Language often spoken at home (LOSH) [1]	6,040 (3.6%)
Knowledge of Official Languages (KOL) [1, 2]	128 (0.4%)
First Official Language Spoken (FOLS) [1, 3]	10,036 (6.0%)
Language spoken to the doctor	2,984 (1.8%)
Language of interview	3,828 (1.9%)
Language of preference	3,811 (1.9%)
**Administrative health data**	
Primary language spoken at home at regular basis – CCRS (*n* = 212,954)	6,883 (3.2%)
Primary language – HCRS (*n* = 716,698)	19,854 (2.8%)
Primary language – OMHRS (*n* = 233,408)	3,146 (1.4%)

CCHS: Canadian Community Health Survey, CCRS: Continuing Care Reporting System, HCRS: Home Care Reporting System, OMHRS: Ontario Mental Health Reporting System

^1^ Include those who speak English and French

^2^ Only available for cycle 2011/2012

^3^ Redefined for cycles 2003 to 2009, based on Statistics Canada definition of FOLS introduced in 2011 (Ref. 25)

The analysis of the levels of concordance between the two data sources (self-report surveys and administrative health databases) showed that the language variables in the health data from home care and long-term care had the highest agreement with LOSH (kappa = 0.76 [0.73–0.79] and 0.75 [0.70–0.80], respectively) (Table 3). The language variables from these two databases (HCRS and CCRS) also held a high level of agreement with FOLS (kappa = 0.66 [0.61–0.71] for both). The language variable in OMHRS (mental health) had poor agreement with the language variables from survey data.

**Table 3:** Agreement analysis for identifying Francophones for matching individuals in the survey (CCHS) and are in administrative health databases (kappa statistic, [95%CI]) (*n* = 27,111)

Language variables in CCHS	Francophones language variables in health data		
	Long-term care - CCRS (*N* = 5639)	Home care – HCRS (*N* = 17,760)	OMHRS (*N* = 3712)
Mother tongue	0.611 (0.564–0.658)	0.607 (0.579–0.633)	0.360 (0.288–0.431)
Language often spoken at home (LOSH)	0.750 (0.70–0.80)	0.764 (0.735–0.793)	0.540 (0.440–0.639)
Knowledge of official languages (KOL)*	0.421 (0.230–0.636)	0.284 (0.271–0.298)	-
Language spoken to doctor	0.678 (0.634–0.722)	0.608 (0.567–0.647)	0.456 (0.329–0.574)
First official language spoken (FOLS)	0.662 (0.613–0.712)	0.665 (0.636–0.693)	0.360 (0.282–0.438)

CCHS: Canadian Community Health Survey, CCRS: Continuing Care Reporting System, HCRS: Home Care Reporting System, OMHRS: Ontario Mental Health Reporting System

*KOL was only available in the 2011/2012 cycle (-) No valid records for estimation

**Fig. 2:**
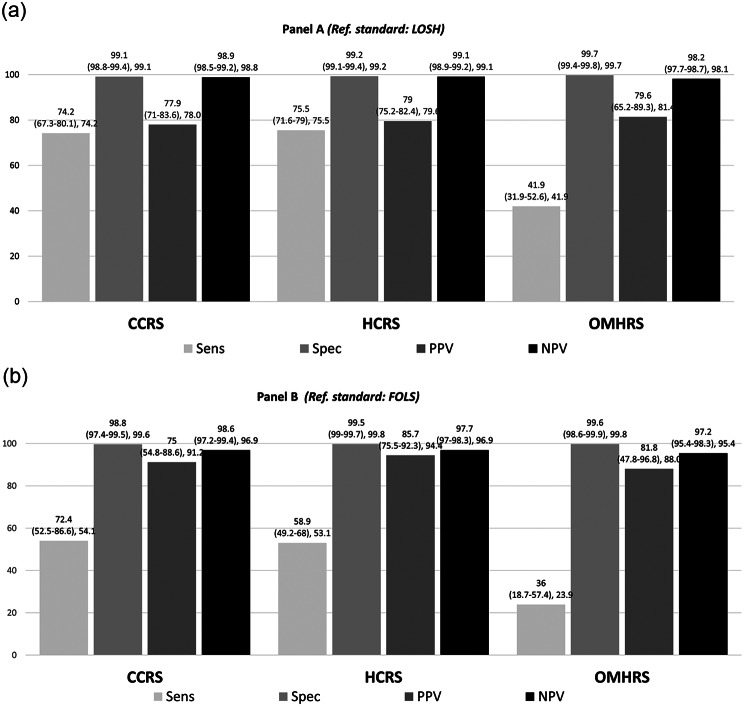
Sensitivity, specificity and predictive values of language variables in administrative health data (*n* = 27,111). Sens: sensitivity; Spec: specificity; PPV: positive predictive value; NPV: negative predictive value, CCRS: Continuing Care Reporting System, HCRS: Home Care Reporting System, OMHRS: Ontario Mental Health Reporting System

When comparing language variables from administrative health databases to self-reported data, we found that the language variables from home care and long-term care databases (HCRS and CCRS) were modestly sensitive (53.1% [49.8–56.4] and 54.1% [48.3–59.7], respectively) but highly specific (99.8% [99.7–99.9] and 99.6% [99.4–99.8], respectively) when FOLS was used as the reference standard. Furthermore, these variables also had very high PPVs (94.4% [92.0-96.2] and 91.2% [85.9–94.7], respectively) and NPVs (96.9% [96.3–97.3] for both data sources) (see Fig. 2 and Table S3 in supplementary material). The sensitivity was even higher when LOSH was used as the reference standard (75.5% [71.6–79.0] and 74.2% [67.3–80.1] for HCRS and CCRS, respectively). The predictive values were also very high with this reference standard for both the HCRS and CCRS databases (PPV 79.6% [75.2–82.4] and 78.0% [71.0-83.6], respectively; and NPV of 99.1% [98.9–99.2] and 98.8% [98.5–99.2], respectively) (Fig. 2).

Consistent with the agreement analysis, the language variable from OMHRS had very low sensitivity (23.9% [18.1–30.9]) against LOSH but very high specificity (99.8% [99.5–99.9]) and high predictive values (PPV and NPV). Details of all estimations are provided in Table S4, Appendix 3.

## Discussion

This study sought to assess the validity of language variables in administrative health databases by comparing language variables recorded in these databases to language data from the CCHS (i.e., LOSH and FOLS), which was taken to be the reference standard. Agreement and validity analyses were carried out, with the objective of identifying Francophones in Ontario in administrative data. Language variables from home care and long-term care data had the highest level of agreement with LOSH and FOLS, while the language variable from mental health admissions had poor agreement with the language variables in the CCHS.

While “primary language” is the language variable most commonly used to collect information in healthcare settings [8, 38, 39], the definition varies across databases and across the healthcare literature; some studies define primary language to be analogous to the language most commonly used (e.g., at home, at school, at work) [8, 40], while others consider the respondent’s first language learned (or mother tongue) to be their primary language [41–43]. The results of this study suggest that the linguistic concept captured by the language variables in both home care and long-term care databases is most similar to LOSH, which showed the highest level of agreement of the language information from home care and LTC settings (kappa = 0.764 and 0.75, respectively). Health care professionals who perform the interviews for home care using the HCRS are encouraged to “observe and listen” to the patient and their family to identify the patient’s primary language and to determine the need for an interpreter [44]. Thus, it is not surprising that the language variable in home care and long-term care databases (HCRS and CCRS) corresponds to the language that the patient most commonly uses to communicate in their own home. This definition of primary language (i.e., language most commonly used either at home or on a day-to-day basis) is similar to that used in previous healthcare studies performed with administrative data [45–47]. 

This study found a high level of concordance between language variables in administrative databases (HCRS and CCRS) when using FOLS as the reference standard. There was very high specificity for ascertaining the Francophones comparing administrative health databases to both LOSH and FOLS, but sensitivity was higher when compared against LOSH. These findings suggest that some home care and long-term care recipients who were identified as Francophones in administrative health databases captured those whose LOSH was French but often missed those whose FOLS was French, which is consistent with the finding of a higher proportion of French speakers with FOLS. Furthermore, given that mother tongue, a component of FOLS, captured the greatest number of Francophones, it is likely that FOLS identified Francophones by mother tongue who no longer speak French on a regular basis at home. In addition, given the higher level of bilingualism among francophones, might influence the decision of many of them to report English as the main language when seeking and receiving care in Ontario. This offer francophones some advantage in a linguistic minority context, when services in French are not available or experience of discrimination or lower quality of care.

The very high predictive values for both CCRS and HCRS implied that participants identified as Francophones in the administrative health databases are very likely to have self-identified as Francophones in the CCHS. Coding errors in administrative health databases may partly account for low sensitivity. It is also possible that administrative health data captured individuals who are fully bilingual and comfortable seeking care from English providers (and thus more likely to be coded as Anglophone), whereas survey data may have included more unilingual Francophones (or Francophones with low English proficiency), who are less likely to seek healthcare services in Ontario [48, 49], which are generally provided in English.

Interestingly, the rate of bilingualism is higher among Francophones than Anglophones [50], which is consistent with the finding of very high specificity and very high negative predictive value. In other words, some Francophones were identified as Anglophones, but very few Anglophones were identified as Francophones. Overall, these results highlight the importance of individual language preference for multilingual patients when seeking care, which may depend on the context (e.g., interpreter use, bilingual provider), as shown in other studies [41, 51]. 

Concordance and sensitivity for identifying Francophones were very low for the OMHRS database. The poor concordance for the language variable in the database related to mental health hospitalizations (i.e., OMHRS) may be related to data entry errors and underreporting. Unlike home care and long-term care assessments, which are performed in the outpatient setting, data for OMHRS are collected in acute care settings. As such, it is likely that interviewers spend less time performing assessments for OMHRS because of competing tasks (e.g., admission documents, clinical care) that must also be performed simultaneously. Furthermore, since the OMHRS captures patients admitted to inpatient mental health hospitals, patients may not be able to provide accurate information due to an underlying mental health disorder (e.g., depression, mania, psychosis). In these situations, reported answers may be influenced by an accompanying person or may be assumed by the interviewer. These factors could bias the interviewers to report the patient’s language as English since it is the most common language at most hospitals in Ontario.

Despite the high concordance of primary language captured in administrative health databases with the language reported in survey data, these results do not imply that there is a single approach to identifying linguistic groups. The approach to selecting the most appropriate language variable for a study should be guided by the design and research question of the study [7, 12], since these elements can impact the language concept of interest. For example, researchers examining the impacts of language barriers may choose a variable that identifies people who can and cannot speak a given language, while researchers studying disparities across ethnolinguistic groups may select a variable such as mother tongue to identify all members of the group in question.

The study design should also be taken into account when performing validation studies of language variables in other administrative databases. Researchers should carefully consider the linguistic concept in the context of the proposed research question while also examining the quality of the administrative data to determine the optimal reference standard for validation. For example, FOLS, which creates linguistic groups denoting Canada’s two official languages (English and French), may not be relevant when studying minority groups other than Francophones, which consist of a higher proportion of individuals who speak neither English nor French. In such instances, language variables such as LOSH or mother tongue may be more suitable.

### Strengths and limitations

For this study, two language variables from a self-report survey (CCHS) were used as the reference standard for respondents’ language. This reference standard, which has not been validated to our knowledge, is subject to self-reporting bias since respondents may overestimate or underestimate their language proficiency. However, self-reported data have previously been used in validation studies of other administrative databases [24–26]. Moreover, the CCHS is a nationally representative survey that provides robust cross-sectional estimates of sociodemographic and health characteristics of the Canadian population [52]. Finally, the proportions of Francophones and other linguistic groups by mother tongue, LOSH and FOLS from the CCHS are consistent with those obtained from census data [14]. 

Nevertheless, there may remain response bias in the CCHS, as some bilingual participants may have reported English or French as the language often spoken at home despite speaking both languages on a regular basis. Contextual factors may also influence an individual’s decision to report his or her primary language in administrative health databases. Since English is the most common language in Ontario, Francophones who also speak English may have reported their primary language as English because they perceived this answer to be more favorable (social desirability bias). This factor may have led to an underestimation of the number of Francophones identified by administrative health databases.

### Conclusions and implications

To our knowledge, no previous study has examined the agreement between language variables in survey data and administrative health databases. This study revealed that language variables in administrative health databases of home care and long-term care have a high level of concordance with LOSH and FOLS and, thus, can be used to reliably identify linguistic groups for the purpose of performing research to assess the impact of language factors on health outcomes. However, caution must be exercised when using language variables collected from acute care settings (such as OMHRS), as these variables may be less reliable. These results suggest that the language concept captured by administrative health databases, particularly from home care and long-term care data, is most similar to language spoken at home. Reporting guidelines recommend studies that use routinely collected data report potential measurement error and how measurement error potentially biases the study’s findings [37]. Hence, the findings from this study can be used for this purpose.

### Electronic supplementary material

Below is the link to the electronic supplementary material.

Supplementary Material 1

## Data Availability

The data set from this study is held securely in coded form at ICES. While data sharing agreements prohibit ICES from making the data set publicly available, access may be granted to those who meet prespecified criteria for confidential access, available at www.ices.on.ca/DAS. The full data set creation plan and underlying analytic code are available from the authors upon request, understanding that the computer programs may rely upon coding templates or macros that are unique to ICES and are therefore either inaccessible or may require modification.

## Abbreviations

CCHS: Canadian Community Health Survey
CCRS: Continuing Care Reporting System
FOLS: First official language spoken
HCRS: Home Care Reporting System
KOL: Knowledge of Official Languages
LOSH: Language often spoken at home
LTC: Long-term care
NPV: Negative predictive value
OHIP: Ontario’s universal health insurance plan
OMHRS: Ontario Mental Health Reporting System
PPV: Positive predictive value
RAI-HC: Resident Assessment Instrument-Home Care
