# Supplementary material for: Ascertaining the Francophone population in Ontario: validating the language variable in health data
Source: BMC Med Res Methodol. 2024 Apr 27;24:98. doi: 10.1186/s12874-024-02220-7 (PMC11055282; doi:10.1186/s12874-024-02220-7)
Supplement: Supplementary file 1 — Supplementary Material 1 [file 12874_2024_2220_MOESM1_ESM.docx]

Defining the francophone population in Ontario: validating the language variable in health data in ascertaining the francophone population

**Supplementary Material**

***Appendix 1. Databases used in the study.***

***Appendix 2. Language definitions and sources of the language variables in health data***

- *Box S1. Language definitions*
- *Box S2. Language variables collected in administrative health data.*

***Appendix 3. Supplementary tables***

- *Table S1. Summary of the study sample by language group.*
- *Table S2. Socio-demographic characteristics of Ontarians who completed the CCHS Cycle 1.1 (2000-2001) to CCHS 2012 (weighted population estimates).*
- *Table S3. Total of French-speaking population estimated in the study sample by type of language variable.*
- *Table S4. Validity measures for Francophones using admin health and self-reported data using FOLS and LOSH as reference standard.*

**Appendix 1. Databases used in the study.**

| Dataset | Description |
| --- | --- |
| Canadian Community Health Survey (CCHS) – cycles 2000/01-2012 | The CCHS is a cross-sectional national representative survey that collects information related to health status, health care utilization and health determinants of the Canadian population aged 12 years or older living in private dwellings in all provinces and territories. Individuals on Indian reserves and on crown lands, institutionalized Canadian residents, full-time members of the Canadian Forces, and residents of certain remote regions are excluded from the survey. The annual component of the CCHS includes cross-sectional information about health, health behaviours and health care use of the non-institutionalized household population aged 12 and older. The CCHS focus content surveys are designed to provide cross-sectional, provincial level results on specific health topics.  Statistics Canada asks participants permission to link information collected during the interview, includes linking survey information to past and continue use of health services, such as visits to hospitals, clinics and doctor’s offices, ensuring that the information will be kept confidential and used only for statistical purposes.  Approximately 85% of participants in the CCHS survey cycles have granted their consent to share their data for linking it with other administrative data (<https://www23.statcan.gc.ca/imdb/p2SV.pl?Function=getSurvey&Id=1383236>). |
| Continuing Care Reporting System (CCRS) | CCCRS collects population-based resident information of patients receiving 24-hour nursing care in publicly funded residential long-term care. A detailed clinical assessment containing clinical, functional and resource utilization (placement) information on individuals receiving continuing care services, including long-term care, in Ontario |
| Home Care Reporting System (HCRS) - Resident Assessment Instrument for Home Care (RAI-HC) | RAI-HC collects information on adults expected to receive publicly funded home care services in Ontario for at least six months. A detailed clinical assessment administered by trained case managers at regular intervals (recommended every 6 months) to all long-stay home care (publicly funded) clients in Ontario. Data include clinical, functional and resource utilization information and are used to inform client needs. The assessment is mandated for all long stay home care clients in Ontario, and each assessment is maintained in the RAI-HC database. |
| Ontario Mental Health Reporting System (OMHRS) | OMHRS collects data on patients admitted to inpatient mental health services. It includes adult designated inpatient mental health care facilities, including beds in General, Provincial Psychiatric, and Specialty Psychiatric facilities. |
| Ontario Health Insurance Plan (OHIP) | OHIP database registers claims made by all physicians for services provided to Ontario residents and paid under the Ontario Health Insurance Plan. The data cover services offered by all health care providers, including physicians, groups, laboratories, and out-of-province providers. |
| Registered Persons Database (RPDB) | The RPDB provides basic demographic information about anyone who has ever received an Ontario health card number. Data is supplied by Ontario Ministry of Health. |

**Appendix 2. Language definitions and sources of the language variables**

*Box S1. Language definitions from CCHS*

| Mother tongue: refers to the first language learned at home in childhood and still understood by the person at the time the data was collected.   - Survey question: What is the language that you first learned at home in childhood and can still understand? (list of language options) |
| --- |
| Language spoken most often at home: refers to the language the person speaks most often at home at the time of data collection. A person can report more than one language as "spoken most often at home" if the languages are spoken equally often.   - Survey question: What language do you speak most often at home? (list of language options) |
| Knowledge of official languages: refers to whether the person can conduct a conversation in English only, French only, in both or in neither language.   - Survey question: Of English or French, which language(s) do you speak well enough to conduct a con-versation? Is it... English only, French only, Both English and French, Neither English nor French? |
| Language of interview: refers to the language in which the interview is conducted recorded.   - Survey question: The interviewer records the language of interview (list of language options) |
| Language of preference: refers to the language of preference for the household interview that is reported by the participant.   - Survey question: Would you prefer that I speak in English or in French? English, French, Other. If Other, What language would you prefer?) (list of language options) |
| Language spoken to a doctor: refers to the language the person uses regularly to speak to the doctor. (Question: Do you and this doctor usually speak in English, in French, or in another language?).   - Survey question: You and this doctor usually speak in English, in French, or in another language? (list of language options) |
| First official language spoken: refers to the first Canadian official language (i.e., English or French) learned, still understood and spoken by the person. For the censuses, this variable is derived within the framework of the application of the Official Languages Act. The same method for deriving the variable was applied here. This derivation method is described in the regulations concerning the use of official languages for the provision of public services.   - It takes into account first the knowledge of the two official languages, second the mother tongue, and third the language spoken at home (<https://www23.statcan.gc.ca/imdb/p3Var.pl?Function=DEC&Id=34004>). |

More information available in Canadian Community Health Survey (CCHS): <https://www.statcan.gc.ca/en/statistical-programs/instrument/3226_Q1_V8>

*Box S2. Language variables collected in administrative health data*

| Database | Type of data | Language label | Definition | Data Collection |
| --- | --- | --- | --- | --- |
| CCRS | Long-term care | Primary language spoken | The primary language spoken by the resident at home on a regular basis | **Process**: Code the primary language spoken by the resident at home on a regular basis as per table 4.2.  A list of valid codes is provided in table 4.2; e.g. eng (English), fra (French), chi (Chinese), etc. |
| HCRS | Home Care | Primary  language | The language the client primarily speaks or understands | **Intent**: To record the person’s preferred language for day-to-day communication.  **Process**: Interview the client and family. Observe and listen. Review any clinical records or check with family or referral source to determine the need for an interpreter. List of codes is provided to be registered; e.g. eng (English), fre (French), deu (German), etc. |
| OMHRS | Mental health admissions | Language | The language the person generally prefers to use for day-to-day communication. | **Intent**. To monitor the service utilization for French, English and other languages and to have information about the person’s language in order to provide interpretation services, if necessary.  **Process**. Ask the person or check with the family if the person is uncommunicative. Enter “eng” if the language is identified as English. Enter “fra” if the language is identified as French. For most other languages, refer to the CIHI Standard Language Pick-List, which is a subset of ISO 639-3 language codes.  **Coding**. Refer to the CIHI Standard Language Pick-List. |

Source:

1. interRAI Overview Committee. RAI-Home Care (RAI-HC) User’s Manual. Canadian Version 2010. Canadian Institute for Health Information, Ottawa, Ont. 2010
2. Canadian Institute for Health Information, Continuing Care Reporting System Specifications Manual, 2009. Ottawa, Ont.: CIHI, 2008.
3. Ontario Mental Health Reporting System Resource Manual, 2019–2020. Canadian Institute for Health Information, Ottawa, Ont. 2019

**Appendix 3. Supplementary tables**

*Table S1. Summary of the study sample by language group*

|  | Total population | French-speakers | | Non-French-speakers | |
| --- | --- | --- | --- | --- | --- |
|  | # | # | % | # | % |
| CCHS combined cycles | 198,509 | 10,036  (by FOLS*) | 6.03 | 188,473 | 94.9 |
| Admin health data |  |  |  |  |  |
| CCRS | 212,954 | 6883 | 3.2 | 206,071 | 96.8 |
| RAI-HC | 716,698 | 19854 | 2.8 | 696,844 | 97.2 |
| OMHRS | 233,408 | 3146 | 1.4 | 230,262 | 98.7 |
| Data linkage |  |  |  |  |  |
| CCRS | 5639 | 214 | 3.8 | 5,425 | 96.2 |
| RAI-HC | 17760 | 632 | 3.6 | 17,128 | 96.4 |
| OMHRS | 3712 | 66 | 1.8 | 3,646 | 98.2 |
| Total | **27111** | **912** | **3.4** | **26,199** | **96.6** |

*Table S2. Socio-demographic characteristics of Ontarians who completed the CCHS Cycle 1.1 (2000-2001) to CCHS 2012 (weighted population estimates).*

|  | **Total population** | | **French-speakers (by FOLS*)** | |
| --- | --- | --- | --- | --- |
|  | (N=63,855,400) | | (N=3,228,300) | |
|  | *#* | *%* | *#* | *%* |
| **Age group** |  |  |  |  |
| <18 | 5,841,000 | 9.1 | 210,900 | 6.7 |
| 18-49 | 34,923,400 | 54.7 | 1,570,800 | 49.7 |
| 50-59 | 9,871,100 | 15.5 | 613,900 | 19.4 |
| 60-69 | 6,813,300 | 10.7 | 427,200 | 13.5 |
| 70-79 | 4,356,300 | 6.8 | 244,500 | 7.7 |
| 80-89 | 1,871,800 | 2.9 | 85,400 | 2.7 |
| 90+ | 178,600 | 0.3 | 7,500 | 0.2 |
| **Sex** |  |  |  |  |
| Male | 31,339,300 | 49.1 | 1,462,400 | 45.5 |
| Female | 32,516,100 | 50.9 | 1,750,600 | 54.5 |
| **Urban and Rural Areas** |  |  | 3,165,700 | 5.0 |
| Urban | 54,591,600 | 85.5 | 2,606,600 | 82.3 |
| Rural | 9,263,800 | 14.5 | 559,100 | 17.7 |
| **Immigrant** |  |  |  |  |
| Yes | 19,583,400 | 30.8 | 228,200 | 8.0 |
| No | 44,035,000 | 69.2 | 2,642,600 | 92.0 |
| **Marital status** |  |  |  |  |
| Married | 33,374,100 | 52.3 | 1,675,800 | 52.7 |
| Common-law | 3,829,800 | 6.0 | 313,400 | 9.9 |
| Widowed | 3,015,400 | 4.7 | 179,800 | 5.7 |
| Separated | 1,641,100 | 2.6 | 109,900 | 3.5 |
| Divorced | 2,659,800 | 4.2 | 137,100 | 4.3 |
| Single, never married | 19,288,000 | 30.2 | 763,800 | 24.0 |
| **Highest level/education** |  |  |  |  |
| < Second. School Grad. | 14,071,400 | 22.2 | 822,100 | 25.8 |
| Secondary School Grad. | 11,350,000 | 17.9 | 471,300 | 14.8 |
| Some Post-Secondary | 4,874,600 | 7.7 | 212,100 | 6.7 |
| Post-Secondary Grad. | 33,113,200 | 52.2 | 1,680,200 | 52.7 |
| **Household income** |  |  |  |  |
| Quintile 1 | 7,573,800 | 19.2 | 228,200 | 21.6 |
| Quintile 2 | 7,500,000 | 19.0 | 218,000 | 20.6 |
| Quintile 3 | 7,635,300 | 19.4 | 213,900 | 20.3 |
| Quintile 4 | 8,178,100 | 20.8 | 225,700 | 21.4 |
| Quintile 5 | 5,841,000 | 21.6 | 170,400 | 16.1 |

^*^ Redefined for cycles 2003 to 2009, based on Statistics Canada definition of FOLS introduced in 2011 (Ref. 26)

CCHS: Canadian Community Health Survey

FOLS: First official language spoken

*Table S3. Total of French-speaking population estimated in the study sample by type of language variable*

| **Language variables** | **French speakers** | | | |
| --- | --- | --- | --- | --- |
| **Survey data** *(combined CCHS cycles)* | Unweighted sample size *(n=198,509)* | | Weighed population estimate *(N=63,855,400)* | |
|  | n | % | N | % |
| Mother tongue | 2,530 | 6.3 | 4,030,600 | 6.3 |
| Language often spoken at home (LOSH)^1^ | 6,040 | 3.6 | 1,942,900 | 3.0 |
| Knowledge of Official Languages (KOL)^1, 2^ | 128 | 0.4 | 41,200 | 0.1 |
| First Official Language Spoken (FOLS)^1, 3^ | 10,036 | 6.0 | 3,228,300 | 5.1 |
| Language spoken to the doctor | 2,984 | 1.8 | 959,900 | 1.5 |
| Language of interview | 3,828 | 1.9 | 1,231,400 | 1.9 |
| Language of preference | 3,811 | 1.9 | 1,225,900 | 1.9 |
|  |  |  |  |  |

^1^ Include those who speak English and French.

^2^ Only available for cycle 2011/2012

^3^ Redefined for cycles 2003 to 2009, based on Statistics Canada definition of FOLS introduced in 2011 (Ref. 25)

CCHS: Canadian Community Health Survey

*Table S4. Validity measures for Francophones using admin health and self-reported data using FOLS and LOSH as reference standard.*

| **Measure** | **CCRS** | | | | **HCRS** | | | | **OMHRS** | | | |
| --- | --- | --- | --- | --- | --- | --- | --- | --- | --- | --- | --- | --- |
|  | (Long-term care data) | | | | (Home care data) | | | | (Mental health admissions data) | | | |
|  | ***FOLS*** | | ***LOSH*** | | ***FOLS*** | | ***LOSH*** | | ***FOLS*** | | ***LOSH*** | |
|  | *Point est.* | *(95%CI)* | *Point est.* | *(95%CI)* | *Point est.* | *(95%CI)* | *Point est.* | *(95%CI)* | *Point est.* | *(95%CI)* | *Point est.* | *(95%CI)* |
| Kappa | 0.662 | (0.6134-0.7115) | 0.7502 | (0.7002-0.8002) | 0.664 | (0.6360-0.6929) | 0.7638 | (0.7349-0.7927) | 0.360 | (0.2816-0.4382) | 0.5398 | (0.4403-0.6394) |
| Sensitivity | 54.1 | (48.3-59.7) | 74.2 | (67.3-80.1) | 53.1 | (49.8-56.4) | 75.5 | (71.6-79.0) | 23.9 | (18.1-30.9) | 41.9 | (31.9-52.6) |
| Specificity | 99.6 | (99.4-99.8) | 99.1 | (98.8-99.4) | 99.8 | (99.7-99.9) | 99.2 | (99.1-99.4) | 99.8 | (99.5-99.9) | 99.7 | (99.4-99.8) |
| Positive Predictive Value | 91.2 | (85.9-94.7) | 77.9 | (71.0-83.6) | 94.4 | (92.0-96.2) | 79.6 | (75.2-82.4) | 88.0 | (75.0-95.0) | 81.4 | (65.2-89.3) |
| Negative Predictive Value | 96.9 | (96.3-97.3) | 98.9 | (98.5-99.2) | 96.9 | (96.7-97.2) | 99.1 | (98.9-99.2) | 95.4 | (94.6-96.1) | 98.2 | (97.7-98.7) |
| Positive agreement (%) | 67.9 |  | 76.0 |  | 68.0 |  | 77.2 |  | 37.6 |  | 54.9 |  |
| Negative agreement (%) | 98.2 |  | 99.0 |  | 98.3 |  | 99.2 |  | 97.6 |  | 98.9 |  |
| Observed proportion of agreement | 0.966 |  | 0.981 |  | 0.969 |  | 0.984 |  | 0.953 |  | 0.979 |  |
| Expected proportion of agreement | 0.901 |  | 0.924 |  | 0.906 |  | 0.931 |  | 0.927 |  | 0.955 |  |
| % Franco by admin data | 6.6 |  | 4.1 |  | 6.3 |  | 3.7 |  | 5.9 |  | 3.0 |  |
| % Franco by FOLS/LOSH: | 3.9 |  | 3.9 |  | 3.5 |  | 3.5 |  | 1.6 |  | 1.6 |  |

LOSH: Language often spoken at home

FOLS: First Official Language Spoken

CCRS: Continuing Care Reporting System

HCRS: Home Care Reporting System

OMHRS: Ontario Mental Health Reporting System
